# Regeneration of the immunoglobulin heavy-chain repertoire after transient B-cell depletion with an anti-CD20 antibody

**DOI:** 10.1186/ar1731

**Published:** 2005-04-01

**Authors:** Anne-Sophie Rouzière, Christian Kneitz, Arumugam Palanichamy, Thomas Dörner, Hans-Peter Tony

**Affiliations:** 1 Department of Medicine II, Rheumatology and Clinical Immunology, University of Wuerzburg, Germany; 2 Charité University Hospital, Berlin, Germany

## Abstract

B-cell depletive therapies have beneficial effects in patients suffering from rheumatoid arthritis. Nevertheless, the role of B cells in the pathogenesis of the disease is not clear. In particular, it is not known how the regeneration of the B-cell repertoire takes place. Two patients with active rheumatoid arthritis were treated with rituximab, and the rearranged immunoglobulin heavy-chain genes (Ig-V_H_) were analysed to follow the B-cell regeneration. Patient A was treated with two courses of rituximab, and B-cell regeneration was followed over 27 months by analysing more than 680 Ig-V_H _sequences. Peripheral B-cell depletion lasted 7 months and 10 months, respectively, and each time was accompanied by a clinical improvement. Patient B received one treatment course. B-cell depletion lasted 5 months and was accompanied by a good clinical response. B cells regenerated well in both patients, and the repopulated B-cell repertoire was characterised by a polyclonal and diverse use of Ig-V_H _genes, as expected in adult individuals. During the early phase of B-cell regeneration we observed the expansion and recirculation of a highly mutated B-cell population. These cells expressed very different Ig-V_H _genes. They were class-switched and could be detected for a short period only. Patient A was followed long term, whereby some characteristic changes in the V_H_2 family as well as in specific mini-genes like V_H_3–23, V_H _4–34 or V_H _1–69 were observed. In addition, rituximab therapy resulted in the loss of clonal B cells for the whole period.

Our data show that therapeutic transient B-cell depletion by anti-CD20 antibodies results in the regeneration of a diverse and polyclonal heavy-chain repertoire. During the early phase of B-cell regeneration, highly mutated B cells recirculate for a short time period in both the patients analysed. The longitudinal observation of a single patient up to 27 months shows subtle intraindividual changes, which may indicate repertoire modulation.

## Introduction

Although the role of B cells in autoimmunity is not completely understood, their importance in the pathogenesis of autoimmune diseases has been further appreciated in the past few years. It is now well known that B cells are more than just the precursors of (auto)antibody-secreting cells [1-4]. They are also involved in the regulation of T-cell-mediated autoimmune diseases by different mechanisms. In this regard, B cells are very efficient antigen-presenting cells. Activated B cells express co-stimulatory molecules, such as CD154, and in this way contribute to the evolution of T effector cells. They can produce chemokines and cytokines, like lymphotoxin α/β, that are essential for the differentiation of follicular dendritic cells in secondary lymphoid organs and for the organisation of effective lymphoid architecture.

There are also indications that B-cell activity is enhanced in rheumatoid arthritis (RA) [2,5]. B cells are found in the synovium, where they form aggregates with T cells and develop tertiary lymphoid tissue structures [5]. The mutational activity of these B cells is markedly enhanced and abnormalities in positive selection and negative selection are found [2]. Furthermore, elimination of B cells by anti-CD20 antibodies from the synovial tissue provokes a disruption of T-cell activation and provokes the production of proinflammatory monokines [6], proving an important role of B cells in the pathogenesis of RA.

The B-cell repertoire is shaped by a complex set of gene rearrangements, somatic hypermutation and receptor-driven selection. These processes are highly regulated during development, ontogeny and the response to antigen [7]. B cells develop in the bone marrow and in the foetal liver, and they mature in the peripheral lymphoid organs. The immunoglobulins they produce contain two heavy polypeptide chains and two light polypeptide chains. The different gene segments are assembled together during recombination to produce a unique rearrangement. The diversity of the repertoire is increased by the addition of or the deletion of nucleotides at the junction between the different gene segments and by random pairing of the heavy chains and light chains. Additional diversity is created by somatic hypermutation, which introduces point mutations to change amino acid codons. This final event takes place in germinal centres, when B cells encounter antigen. The composition of the antibody repertoire is regulated and constrained, and there is substantial evidence that the B-cell repertoire is changed in autoimmune diseases, such as systemic lupus erythematosus [1], Sjögren's syndrome [8,9], myasthenia gravis [10], diabetes mellitus [11,12] or RA [13,14].

B-cell depletive therapies have beneficial effects in patients suffering from RA [14-22]. Rituximab is a chimeric anti-CD20 monoclonal antibody that consists of human IgG_1 _and kappa constant regions and of mouse variable regions from a hybridoma directed at human CD20. Rituximab has mainly been used for the treatment of non-Hodgkin lymphomas [23]. It selectively depletes CD20^+ ^B cells from the peripheral blood, the spleen and the bone marrow for several months [18]. Because early B-cell precursors do not express CD20, the bone marrow is able to repopulate B lymphocytes after therapy.

The aim of the present study was to determine whether a polyclonal and diverse B-cell repertoire is regenerated after temporary B-cell depletion by anti-CD20 monoclonal antibodies. To address this issue, we have analysed the immunoglobulin heavy-chain gene (Ig-V_H_) repertoire of two patients suffering from active RA before and after treatment with rituximab, up to a time period of 27 months.

## Materials and methods

### Patients

A 46-year-old male patient (patient A) was diagnosed with RA using the American College of Rheumatology criteria. The patient was unsuccessfully treated over a time period of 8 years with three disease-modifying anti-rheumatic drug regimens including methotrexate, and also failed therapy with tumour necrosis factor alpha blockers. After giving informed consent, the patient was treated in an open-label protocol, which was approved by the local ethics committee.

Rituximab (Mabthera^®^; Hoffmann La-Roche, Grenzach-Whylen, Germany) was administered intravenously at a dose of 375 mg/m^2 ^once a week for a total of four infusions (days 1, 8, 15 and 22). The disease eventually relapsed 15 months after the beginning of the study. The patient therefore received another rituximab treatment (four once-weekly doses of 375 mg/m^2^) at the time point of 17 months. Except for 5 mg prednisolone equivalent daily, the patient received no other antiproliferative treatment during the whole study.

Patient B was a 63-year-old female patient with rheumatoid factor (RF)-positive RA. She had been unsuccessfully treated over a time period of 7 years with three different disease-modifying anti-rheumatic drug regimens including methotrexate. The patient has not yet been treated with a tumour necrosis factor alpha blocking therapy. After informed consent, she was treated with rituximab according to the same protocol as patient A. In addition, patient B continued to receive 20 mg methotrexate weekly. She did not receive any glucocorticoids during the study.

The analysis of lymphocyte subsets by immunofluorescence staining was performed by incubating peripheral blood mononuclear cells (PBMCs) with anti-CD19 and anti-CD3 antibodies (Phycoerythrin (PE) or Fluorescein isothiocyanate (FITC)-labelled as indicated; all antibodies from Becton-Dickinson, Heidelberg, Germany) using a FACSCalibur (Becton-Dickinson, San Jose, CA, USA). Frequencies of cell populations were calculated using CellQuest software.

Disease activity was regularly determined using the disease activity score (DAS28 index) and by monitoring C-reactive protein (CRP) levels.

### Amplification of rearranged immunoglobulin V genes by PCR

Genomic DNA was isolated from PBMCs using the QIAamp^® ^DNA Blood Mini Kit (Qiagen, Hilden, Germany). Rearranged V_H_DJ_H _gene rearrangements were amplified for all V_H _families using a nested PCR approach [24]. Genomic DNA was amplified in separate reactions for the six V_H _families (V_H_1 oligonucleotide primers also co-amplify perfectly V_H_7 gene rearrangements). The final concentrations of the reagents were 200 μM each dNTP (Peqlab, Erlangen, Germany), 0.625 μM each primer, 2.5 mM MgCl_2_, 10 × PCR buffer II and 2.5 U AmpliTaq DNA polymerase (Applied Biosystems, Foster City, CA, USA).

During the external amplification round, 250 ng (5 μl) DNA were amplified in a 75 μl reaction containing primers specific for the leader peptide sequence and a mixture of external J_H _primers in a Gene Amp PCR System 2400 (Perkin Elmer, Applied Biosystems, Foster City, CA, USA). The internal amplification round was conducted with the 5' primer specific for framework region FR1 and a mixture of internal J_H _primers using 5 μl of the product of the first amplification reaction as template. The cycling parameters have been described previously [24]. Briefly, the annealing temperatures were 50°C for the external amplification round and 65°C for the internal round.

The polymerase error rate for the amplification of V_H _genes using nested PCR has been documented to be 1 × 10^-4 ^mutations/bp [25].

### RT-PCR

Total cellular RNA was extracted from 1 × 10^7 ^PBMCs after lysis of the cells in 1.5 ml TRIZOL reagent (Gibco, Karlsruhe, Germany) following the manufacturer's instructions.

The RT-PCR reaction was performed using Titan One Tube RT-PCR system (Roche, Mannheim, Germany). First-strand cDNA was synthesised at 42°C for 60 min in a 50 μl reaction mix containing 5 mM dithiothreitol, 400 ng oligo-dT_15_, 200 μM dNTP, 8 U RNAse inhibitor, 5 × RT-PCR buffer, 20 U high-fidelity enzyme mix RT-AMV and 1 μg RNA. V_H _mRNA transcripts were amplified by the nested PCR protocol described earlier using 5 μl cDNA and C-region primers (Cμ, TCA GGA CTG ATG GGA AGC CC; Cγ, CGA GCC GCT GGT CAG AGC G; Cα, ACC CTC AGC GGG AAG ACC TT) as the 3' primers in the first round.

### Cloning of rearranged immunoglobulin V genes

All PCR products were separated by electrophoresis through 1.5% agarose and were visualised with ethidium bromide. Successful amplifications were identified as a band corresponding to a product of approximately 350 bp. The bands were excised and subsequently purified using the MinElute Gel extraction kit (Qiagen). The purified PCR products were polished using the PCR polishing kit (Stratagene, Amsterdam Zuidoost, The Netherlands), were ligated with Zero Blunt pCR-blunt vector and were transformed into One Shot^® ^TOP10 cells (Invitrogen, Karlsruhe, Germany)

### Sequencing and analysis of rearranged V genes

Plasmid DNA from clones containing gene inserts was prepared using the Wizard Plus SV Minipreps DNA Purification System kit (Promega, Mannheim, Germany). The DNA sequences were determined using BigDye Terminator Cycle Sequencing Ready Reaction kit (Perkin Elmer, Applied Biosystems) and the M13 forward and reverse universal primers in an automated genetic analyser ABI PRISM 310 (Applied Biosystems). Germline immunoglobulin V genes were identified by blast searching the VBase Sequence Directory [26].

### Single cell sorting and PCR amplification

The procedure for single cell sorting and subsequent PCR amplifications has been described previously [24]. Briefly, single CD19^+^CD27^- ^and CD19^+^CD27^+ ^cells were sorted into wells of 96-well plates using a FACStar Plus flow cytometer with an automated single cell deposit unit (Beckton-Dickinson, USA). After primer extension pre-amplification, the rearranged V_H_DJ_H _genes were amplified by nested PCRs using the same oligonucleotides as those already described. After gel purification (Qiagen), the PCR products were directly sequenced using the BigDye Terminator Cycle Sequencing Ready Reaction kit (Perkin Elmer, Applied Biosystems) and the 5' V primer used for the internal amplification.

### Statistical analysis

The statistical analyses were performed using GraphPad software . Sequences were analysed by the Fisher's exact test to compare the differences in the distribution of particular gene segments. The average lengths of CDR3 were studied by the unpaired *t *test. The chi-square test was used to compare the mutational frequencies.

## Results

### Clinical data

**Figure 1:**
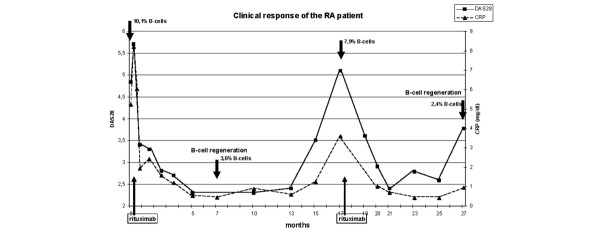
Clinical response of rheumatoid arthritis (RA) patient A. The disease activity score (DAS28 index) and C-reactive protein (CRP) (mg/dl) levels of the RA patient. The patient was treated twice with rituximab (at 0 months and 17 months). Arrows indicate the percentage of B cells detected in peripheral blood for the four time points analysed (0, 7, 17 and 27 months)

Figure 1 shows the clinical response of RA patient A during the two treatment periods with rituximab. B cells accounted for 10.1% of peripheral lymphocytes at the beginning of the study. A rapid B-cell depletion occurred after each therapy, and lasted up to 7 months and 10 months, respectively. The time points of 7 months and 27 months were the first times where B cells were detectable in peripheral blood, either by flow cytometry or by PCR (data not shown). The B-cell frequency was 3% of total lymphocytes at 7 months and was 2.4% of total lymphocytes at 27 months (Fig. 1). Patient A had an active disease before therapy, as indicated by a high CRP level and a high DAS28 clinical activity index. The disease activity declined continuously after rituximab therapy. The patient had a good clinical response starting from 3 months and lasting over 1 year. We observed a deterioration of the clinical parameters about 13 months after the first therapy. The patient therefore received a second treatment with rituximab at 17 months. Again, patient A presented a clinical improvement following B-cell depletion. The arrows in Fig. 1 indicate the four time points when the B-cell repertoire was studied. Table 1 presents the RF values. The RF activity declined quite rapidly after rituximab therapy and followed the inflammatory activity, with increasing values in relapse and falling values after the second treatment.

Patient B started with B cells at 11% of the peripheral blood lymphocytes. B cells were not detectable during the 5 months following the rituximab treatment. B cells represented 2.4% of peripheral lymphocytes at the time point of 5 months, and represented 3.1% at 6 months. The patient experienced a good clinical response. The DAS28 declined from 6.0 before therapy to 3.3 at 5 months, and the CRP values declined from 1.9 mg/dl to 0.65 mg/dl, respectively.

### Distribution of V_H _genes in patient A (Fig. 2)

Immunoglobulin V_H _gene rearrangements from peripheral blood B cells of patient A were analysed using nested PCR, followed by subcloning and sequencing at the time points indicated in Fig. 1. A total of 687 clones were analysed: 179 clones before treatment, 199 clones during the first phase of B-cell regeneration (7 months after the first therapy), 149 clones after 17 months, and 160 clones after 27 months (at the time of the second B-cell regeneration). Only the productive rearrangements were taken into consideration.

#### V_H_1 family

V_H_1–69 and V_H_1–18 were the most frequently used V_H_1 family members before therapy, comprising 68% of the sequences analysed in this family. Significant changes in the V_H_1 distribution could be observed 7 months after therapy. V_H_1–02 was increased and became the predominant gene (36% versus 4%, *P *= 0.0038), whereas V_H_1–69 was decreased (12% versus 36%, *P *= 0.0594). The V_H_1 gene distribution 17 months after therapy was largely comparable with that before treatment (i.e. V_H_1–18 and V_H_1–69 were the most often used genes). Notably, V_H_1–03 was increased and was then the third most frequent gene (18%). The distribution of the V_H_1 genes after 27 months was quite stable with no significant differences to the previous time point, except that V_H_1–03 was no longer found.

#### V_H_2 family

The gene V_H_2–05 was slightly predominant in the V_H_2 family before treatment. The distribution was completely shifted toward the usage of V_H_2–05 7 months after therapy (96% versus 59%, *P *= 0.0041), which 10 months later shifted back to the rearrangement frequency found before treatment. At the time point of 27 months, during the second regeneration phase, the frequencies of the V_H_2 genes were similar to those observed during the regeneration phase following the first treatment (7 months after therapy).

#### V_H_3 family

The V_H_3 family is the largest family, comprising 22 members. Ten family members were found before treatment, two of them accounting for 45% of all V_H_3 rearrangements (V_H_3–23, 29%; V_H_3–30/3–30.5, 16%). The newly regenerated B cells used a greater variety of genes 7 months after therapy (16 different V_H_3 gene segments were observed). The overall distribution was similar to the first time point, except for V_H_3–07, which was then over-represented (15% versus 2%, *P *= 0.059). The gene V_H_3–23 was significantly increased 17 months after therapy when compared with the previous time point (37% versus 17%, *P *= 0.0481), and was the most often represented gene, followed by V_H_3–09 (16% of all V_H_3 rearrangements). No significant alterations in V_H_3 gene distribution could be observed in the final time point, despite the tendency for V_H_3–23 to decrease to its level found at the time point of 7 months.

#### V_H_4 family

One single gene in the V_H_4 family (V_H_4–34) was overexpressed before therapy, accounting for more than 40% of all V_H_4 rearrangements. The therapy induced some significant changes in the distribution of the V_H_4 genes. The frequency of V_H_4–34 decreased (16% versus 41%, *P *= 0.0198), whereas V_H_4–04 increased (21% versus 3%, *P *= 0.0302). V_H_4–59 was also increased and became the most frequently rearranged gene (39% of the V_H_4 sequences after therapy). As described for the V_H_3 family, there was also a greater variety of genes used in the V_H_4 rearrangements after therapy. Seven different gene segments were used before treatment, whereas nine different gene segments were found after treatment. The overall rearrangement frequency of the different genes at the time point of 17 months was comparable with that observed before therapy, except for certain genes like V_H_4–34 that remained at the level seen directly after therapy. Significant changes could be described between the points of 7 months and 17 months after therapy. An increased frequency of the genes V_H_4–39 and V_H_4–30.1/4–31 was observed (18% versus 3%, *P *= 0.0443 and 24% versus 5%, *P *= 0.0372, respectively), as well as a decreased use of the genes V_H_4–59 and V_H_4–04 (12% versus 39%, *P *= 0.0146 and 6% versus 21%, *P *= 0.0931, respectively). This distribution was then quite stable up to 27 months.

#### V_H_5 family

**Figure 3:**
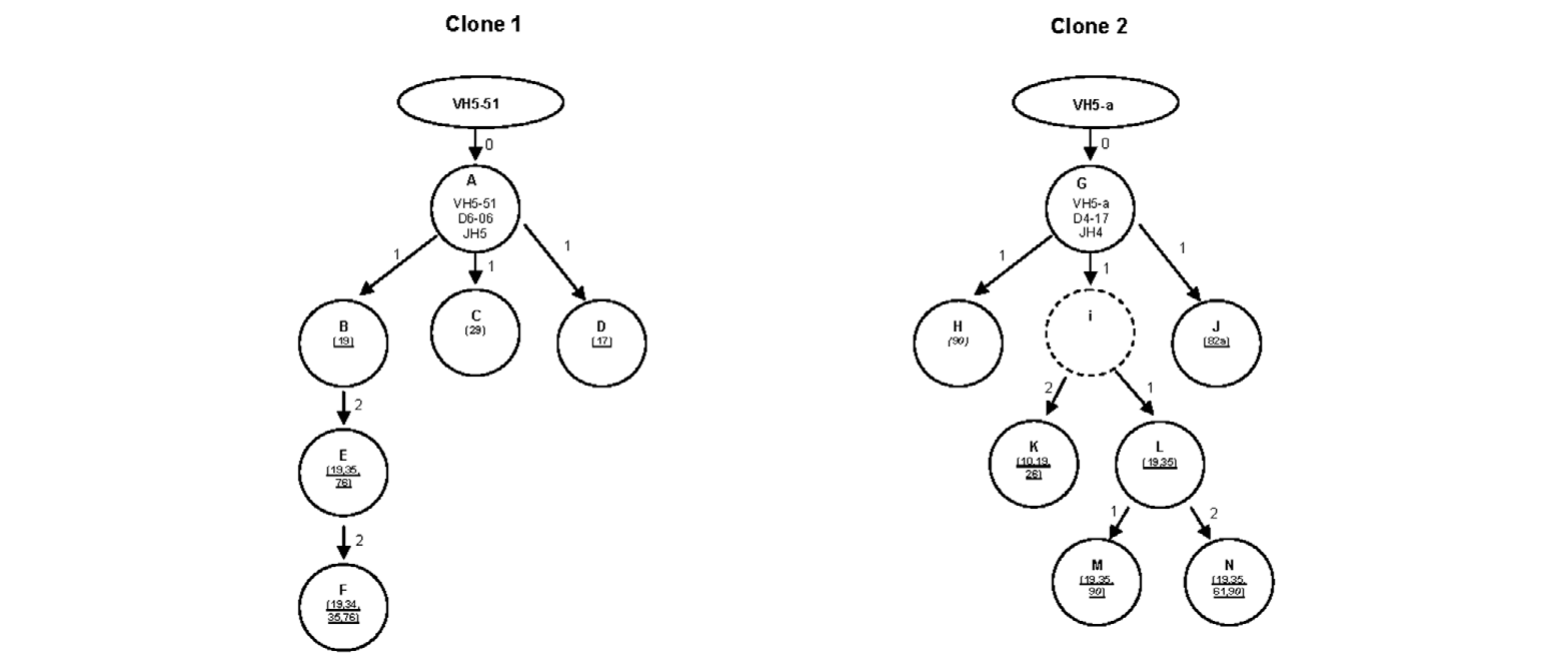
Genealogical trees of B-cell clones found before therapy in V_H_5 family in patient A. The best matching germline V_H _gene segments are shown in ellipses. The letters in the circles refer to individual sequences. Upper circle, parental clones with the gene segments they are using. Dotted circles, deduced intermediates. The numbers alongside the arrows represent the number of mutations between the different sequences. Brackets, mutated codons; underlined, replacement mutations; italicised, mutation to stop codon.

Within the two-member V_H_5 family, V_H_5–51 was the predominant gene at all four time points analysed. Its frequency significantly increased after the first therapy (86% versus 65%, *P *= 0.036), however, with a further tendency to fall to the pretreatment level. In this family, two B-cell clones were found in the repertoire before therapy (Fig. 3). The first clone comprised six clonally related sequences, with the number of shared mutations varying from zero to six per sequence. This first clone's 33-nucleotide CDR3 involved V_H_5–51 rearranged to D6-6 and J_H_5. The second clone consisted of seven clonally related sequences (from which three were nonproductive since one mutation in position 90 generated a stop codon). The CDR3 was 30 nucleotides long. It was composed by V_H_5-a rearranged to D4–14 and J_H_4, and had between zero and four mutations. Both B-cell clones disappeared after the first anti-CD20 therapy; their rearrangements were no longer observed and no other B-cell clones were detected during follow-up.

### Use of D segments, distribution of J_H _gene segments and CDR3 length in patient A

All D gene families could be detected in the sequences analysed. Before treatment, the four-member D1 family was under-represented compared with its representation in the genome (3% versus 16% expected). On the contrary, the D6 family that comprises only three members was over-represented (31% versus 15%). A significant increase of the D1 family members was observed after therapy, bringing the frequency to its expected level. Its usage decreased continuously until 27 months. In parallel, D3 became the most frequently used family starting from 17 months after therapy.

The analysis of the J_H _segments indicated that the overall distribution of these components did not vary with the treatment. J_H_4 was represented most frequently (accounting for 50% or more of all rearrangements), followed by J_H_6 (between 20% and 30% of all rearrangements). The other genes were less frequently used. A significant reduction of the J_H_6 use (20% versus 30%, *P *= 0.0223), as well as a significant increase of J_H_2 (7% versus 1%, *P *= 0.0069), was observed after the first B-cell depletion. The rearrangement frequency of these two gene segments returned to the pretreatment level at 17 months. No significant changes were observed in the other families.

The CDR3 length was calculated by determining the number of nucleotides from residues 95–102. The average length of CDR3 before therapy was 38.0 nucleotides (± 11.0), ranging from 9 to 75 nucleotides. After the first anti-CD20 treatment (7 months after therapy) the average length was 36.9 nucleotides (± 9.4), ranging from 15 to 69 nucleotides; 17 months after therapy the average length was 42.6 nucleotides (± 12.1), ranging from 15 to 78 nucleotides; and at the time point of 27 months the average length was 42.3 nucleotides (± 10.7), ranging from 18 to 75 nucleotides.

### Mutational frequencies in V_H _rearrangements in both patients (Fig. 4)

**Figure 4:**
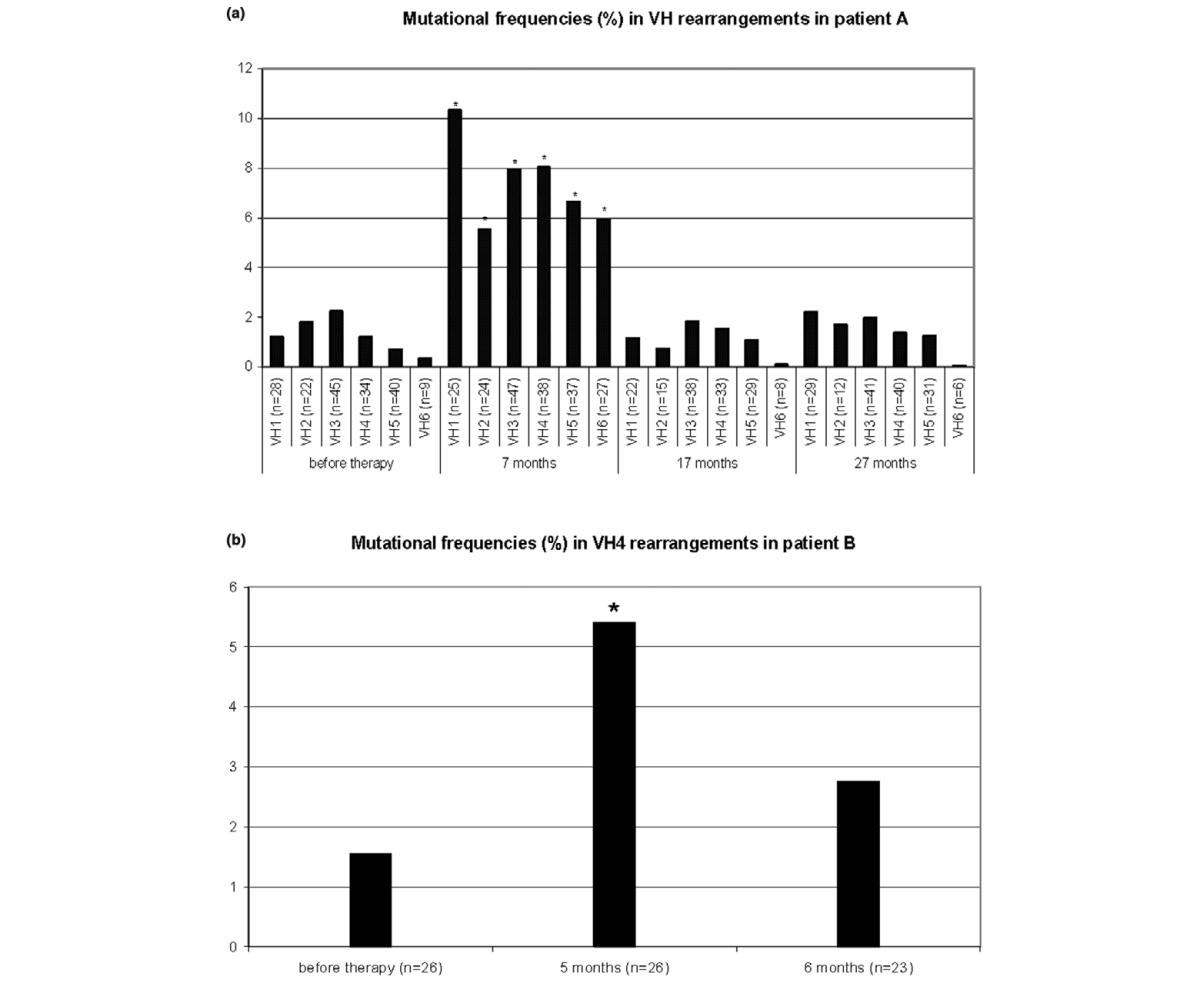
Mutational frequencies in V_H _rearrangements in **(a) **patient A and **(b) **patient B. * *P *< 0.0001 using the chi-square test.

At the beginning of the study, the overall mutational frequency in the V_H _genes of patient A was 1.4% (681 mutations/48,891 bp) (Fig. 4a). The mutational frequencies varied from nine mutations/2,448 bp (0.4%) for the V_H_6 family to 278 mutations/12,390 bp (2.2%) for the V_H_3 family. The majority of the clones (113 out of 178) contained two mutations or less per rearrangement (data not shown). During the first B-cell regeneration phase, the mutational frequencies varied from 383 mutations/6,940 bp (5.5%) for the V_H_2 family to 638 mutations/6,542 bp (9.8%) for the V_H_1 family. The overall mutational frequency was highly and significantly increased at this time point (4,032 mutations/54,720 bp [7.4%] versus 681 mutations/48,891 bp [1.4%] before therapy, *P *< 0.0001). Almost 90% of the sequences analysed (175 out of 198) had more than 10 mutations per sequence. The frequency of mutations at the time point of 17 months was decreased again to the level found before treatment (519 mutations/39,307 bp [1.4%]) and 92 sequences out 145 contained two mutations or less per rearrangement. The overall mutational frequency stayed in the same low range at the later time points up to 27 months (725 mutations/44,037 bp [1.6%]). Only the distribution of the mutations per rearrangement was somewhat distinct, since 24 out of 159 sequences (15.1%) contained more than 10 mutations (versus 10.1% and 9.7%, before and 17 months after therapy, respectively).

The second patient (patient B) was analysed for mutational frequencies in the Ig-V_H _repertoire. The results for the V_H_4 family are presented in Fig. 4b. The mutational frequency before therapy was 1.5% (110 mutations/7,148 bp). Only two sequences out of 26 presented more than 10 mutations per sequence. At the early regeneration point (5 months after therapy), the frequency of mutations was significantly increased to 5.4% (394 mutations/7,292 bp, *P *< 0.0001) and the majority of the sequences (15 out of 26) contained more than 10 mutations. Four weeks later, the mutational frequency was decreased to 2.8% (169 mutations/6,151 bp). The number of sequences containing more than 10 mutations (five out of 23) was lower than in the previous time point but was still elevated compared with that before therapy.

### Overall mutational frequencies of V_H _rearrangements from single CD19^+ ^cells in patient A (Table 2)

In order to substantiate the unexpected high mutation rate observed 7 months after the first B-cell depletion, PBMCs of patient A were sorted into single CD19^+ ^cells that were either CD27^+ ^or CD27^-^. This different approach also revealed very high mutational frequencies in both B-cell populations. The overall mutational frequencies of CD19^+^CD27^- ^single cells before therapy were as low as expected (0.6%). At the time point 7 months, during the first regeneration phase, the mutational frequency was significantly elevated in both CD27^- ^B cells (5.3%) and CD27^+ ^B cells (8.3%).

## Discussion

The implication of B cells in the pathogenesis of RA is now well established, but their precise role is still unknown. Numerous studies have shown that B-cell depletion by anti-CD20 therapy can be beneficial for patients suffering from RA [15,16,19-22]. However, it is not known how the B-cell repertoire regenerates after anti-CD20-mediated transient B-cell depletion. In particular, whether a polyclonal and diverse repertoire is reconstituted has not been studied. To address this question, we decided to compare the B-cell repertoire of a RA patient before and after effective clinical B-cell depletive therapy by analysing his Ig-V_H _repertoire over a time period of 27 months.

A patient with active RA was selected for B-cell depletion using rituximab. He showed a good clinical response for over 1 year after antibody treatment. The disease eventually relapsed and the patient was retreated with rituximab. The second B-cell depletive therapy again induced a significant clinical response lasting about 10 months.

At the beginning of the study, the Ig-V_H _repertoire of the RA patient basically resembled the published distributions for healthy people [24,27]. Nevertheless, certain genes already described with bias in autoimmune diseases were used in a different proportion. In particular, the genes V_H_1–69 and V_H_4–34 were over-represented. The gene V_H_1–69, which represented 35.7% of the rearrangements for the V_H_1 family in the present study, has been found at the frequency of 11.1% in healthy persons [24]. The gene V_H_4–34 represented 41.2% of the V_H_4 genes in our RA patient. Its frequency in healthy people has been described as 14.3% [24] and 15.7% [27]. On the other hand, the gene V_H_3–07 was found in a smaller proportion (2.2% of the V_H_3 genes versus 7.5% [24] and 10.8% [27] in healthy controls). These genes have been shown to exhibit some evidence for (auto)antigen selection; for example, for RF activity [8]. In particular, the gene V_H_4–34 is very often used by anti-DNA antibodies [28,29] and is exclusively used by cold agglutinins [30]. In agreement with Huang and colleagues' data [13], the gene V_H_3–30 was found less frequently in our RA patient than in the controls. In addition to these genes, the proportion of some other variable genes like V_H_1–02, 1–18 or 4–04 also differed from the published data of normal controls and provided a distinct pattern for this patient.

The analysis of D segments and J_H _genes showed distributions comparable with those described in healthy individuals [24,27,31]. D6 represented the predominant D family and J_H_4 was the most frequently used J_H _segment, followed by J_H_6. The only difference we detected before therapy was the under-representation of the D1 family. The CDR3 length average (38.0 ± 11.0 nucleotides) was in agreement with published data [32].

These observations suggest that the overall representation of individual V_H _genes in peripheral B cells in the present RA patient resembled the repertoire expected in an adult, but also contained characteristic differences in certain genes already seen to often be biased in autoimmune diseases.

B cells regenerated well after B-cell depletion, and showed a diverse and polyclonal repertoire. Nevertheless, changes in the Ig-V_H _genes were detected, with the most profound effects observed 7 months after the beginning of therapy, during the early regeneration phase. The intraindividual long-term changes were more subtle.

Seven months after the first therapy was the earliest time point when peripheral B cells could be detected either by flow cytometry or by PCR. These early regenerated B cells presented a distribution of Ig-V_H _genes significantly different from that before therapy. Some V_H _genes (such as V_H_1–02 or V_H_3–07) were more often used, whereas other genes (e.g. V_H_4–34) were decreased. Also, significant changes were observed in the distribution of the D segments and J_H _genes. The most striking differences were the mutational frequencies found in the V_H _genes (Fig. 4a). At this time point, 3% of the peripheral lymphocytes were CD19^+ ^B cells. All the amplified sequences were extensively mutated (mean mutation rate, 7.4%): 88% contained more than 10 mutations per sequence. This was highly significant when compared with the data observed before therapy and at the time point of 17 months, where only 10% of the rearrangements comprised more than 10 mutations per sequence. This increase of mutations correlated well with the diminution of J_H_6 usage and the tendency of lower CDR3 length, and argues the influence of antigen contact and T-cell help [8,31].

The high mutation rates were unexpected. This result is not likely to be related to selective amplification of specific sequences by our PCR protocol, since the high mutation rates were observed in all V_H _families amplified using different PCR conditions. Furthermore the detected repertoire was polyclonal, and even presented an extended number of V_H _genes. We nevertheless wanted to substantiate this result using a different approach. We therefore sorted single cells from this time point in CD27^+ ^and CD27^- ^B-cell subpopulations. The increase of mutational frequency was confirmed: again, the newly recirculating B cells showed increased mutation rates. This was detectable in both CD27^+ ^and CD27^- ^B cells (8.3% for CD27^+ ^and 5.3% for CD27^- ^versus 0.6% for the CD27^- ^cells before therapy; Table 2). The fact that even the CD27^- ^B cells were highly mutated was surprising, since CD27 is assumed to be a marker for memory B cells with mutated immunoglobulin receptors [33,34]. Mutated CD27^- ^B cells have, however, been described in a study by Hansen and colleagues in patients with Sjögren's syndrome [35]. Reparon-Schuijt and colleagues also described, in the synovium of RA patients, a population of B lymphocytes that were functionally and phenotypically distinct from classic memory cells [36]. These cells were CD20^+^, CD38^- ^and CD27^-^, and they produced immunoglobulins under induction but had a defective proliferative responsiveness.

To determine the heavy chain class distribution, we performed RT-PCR on total RNA from this time point (7 months), using primers specific for IgM, IgG and IgA. The IgM population was slightly mutated as expected (1.7%), but the IgG (9.0%) and IgA (8.9%) populations were highly mutated, in the same range as the genes amplified from genomic DNA (7.4%). This is in line with the assumption that the regenerating B cells at this time point were class-switched B cells.

To address the question of whether the circulation of highly mutated B cells in the early regeneration phase may be related to the anti-CD20 mediated B-cell depletion, a second patient was studied. We analysed the mutational frequencies in 75 V_H_4 rearrangements before treatment and 5 months and 6 months later, when B cells were again detectable in the periphery (Fig. 4b). The elevated mutation rate of expressed Ig-V_H _genes during the early regeneration phase was confirmed. Before B-cell depletion, similar to patient A, about 8% of the Ig-V_H _sequences were highly mutated. B-cell depletion in the periphery lasted 5 months in patient B. At the time point of 5 months, 2.4% of peripheral lymphocytes were CD19^+ ^B cells and 46% of the analysed Ig-V_H _sequences were highly mutated. As in patient A, the phenomenon seems to be transient since a decrease in the mutation rate was already observed 4 weeks later in patient B. Only 22% of the rearrangements were highly mutated at this time point.

Although our findings are restricted to a small number of patients, the observed changes in the B-cell repertoire are highly probably related to the regeneration of B cells. We did not observe other possible confounding factors. During this phase, the patients had no change in their medication and did not show any clinical signs of infection. Also, the CRP levels did not change during this time period.

It is not known from which B-cell pool peripheral B cells regenerate after an anti-CD20-mediated B-cell depletion. A recent paper using a mouse model for anti-CD20-mediated immunotherapy demonstrates a hierarchy of B-cell sensitivities using rituximab-mediated B-cell depletion [37]. Particularly, germinal centre B cells and marginal zone B cells were more resistant to depletion *in vivo*. The microenvironment, such as resident macrophages, B-cell survival factors or circulatory dynamics of B-cell subsets, influences their sensitivity to anti-CD20-mediated depletion. It therefore seems probable that B-cell regeneration arises from a distorted composition of the mature B-cell compartment. The recirculating B cells during the early regeneration phase seem not to be newly generated cells, but are more probably resident cells that were resistant to the antibody treatment. These cells form the first wave of regenerating cells. Later B-cell repopulation is then taken over by newly produced naive cells. Alternatively, B-cell regeneration changes the local environment in the central immunological organs in a way that plasma cells, which usually home in the tissues or in bone marrow, recirculate in the periphery for a short time period.

Except for the highly mutated population observed during the early regeneration phase, the regenerated B-cell repertoire was overall relatively stable. This is in agreement with the data of Dijk-Hard and Lundkvist that followed the distribution of V_H _gene families in five individuals over a time period of 9 years [38]. A high degree of stability in the V_H_gene family repertoire was described in that paper, except for one individual where a changing pattern was observed that correlated with the presence of RF in serum at one time point.

Our study was not designed to detect specific disease-relevant changes in the repertoire. However, in addition to the treatment-induced reduction in disease activity, a specific decline in RF activity was observed. The patient showed a high RF activity before treatment. This activity decreased rapidly after the first B-cell depletion and rose again in relapse at 17 months. The second rituximab treatment again resulted in a significant fall of RF activity (Table 1). This rapid decrease in RF is in line with a report in a larger series of patients treated with rituximab [39]. The mechanism of a more selective effect on autoantibody production is not clearly understood. Possibly, RF-producing plasma cells are more dependent on the new regeneration from the CD20^+ ^B-cell pool. Since we do not know precisely the Ig-V_H _genes used by RF-secreting B cells, it is not possible to relate RF to the use of Ig-V_H _genes. Nevertheless, there were distinct changes in the Ig-V_H _repertoire that paralleled the decrease in clinical activity – certain genes were shown to fluctuate, for instance the genes of the V_H_2 family or the gene V_H_3–23. The predominant gene of the V_H_3 family, V_H_3–23, was found in a high proportion before therapy, decreased 7 months after therapy, increased again 17 months after therapy, accompanied by a clinical relapse, and decreased again 27 months after the first therapy. It is also interesting to note that the use of J_H_6 segment in the V_H_3 rearrangements as well as the shorter CDR3s correlated with the disease activity – when the disease was active, the J_H_6 segment was found in a higher proportion accompanied by a shorter CDR3.

Regarding the V_H_4–34 gene already described to be frequently used in autoimmune disorders, it was significantly decreased with therapy and its frequency remained low after therapy. Irrespective of the possible pathogenic role of these changes, these results give evidence for a long-term modulation of the V_H _gene repertoire induced by anti-CD20 antibody treatment. Clonal expansion is a characteristic feature of the patients with RA. B-cell clones have been found in peripheral blood [13,14] and in synovial tissue [5,14]. In the present study, we were able to detect two clones within the V_H_5 family before therapy. The rearrangements used by these two clones were no longer observed after therapy at all studied time points up to 27 months. The inducible loss of clonal B cells is also an indication for the modulation of the B-cell repertoire. Even if their specificity is not known, the relevance of clonal B cells in disease activity can be speculated.

## Conclusion

The present study describes the Ig-V_H _repertoire development after transient anti-CD20-mediated B-cell depletion. The results show that therapeutic, even repeated, transient B-cell depletions by anti-CD20 antibodies result in the regeneration of a diverse and polyclonal heavy-chain repertoire. The early phase of B-cell regeneration is characterised by the recirculation of highly mutated B cells during a short time period in both the patients analysed. The longitudinal observation of a single patient up to 27 months indicates subtle intraindividual changes, which cautiously favour the hypothesis of a therapeutic B-cell repertoire modulation.

## Abbreviations

bp = base pair; CDR = complementary determining region; CRP = C-reactive protein; DAS28 = disease activity score; Ig-V_H _= immunoglobulin heavy-chain; PBMC = peripheral blood mononuclear cells; PCR = polymerase chain reaction; RA = rheumatoid arthritis; RF = rheumatoid factor.

## Competing interests

The author(s) declare that they have no competing interests.

## Authors' contributions

A-SR carried out the molecular genetic study, analysed the results and drafted the manuscript. CK participated in the design and coordination of the study, and performed the characterisation of cells. AP was involved in the molecular analysis of the second patient. TD carried out the single cell sorting and was involved in the molecular analysis. H-PT conceived the study, participated in its design and coordination. All authors read and approved the final manuscript.

## Figures and Tables

**Figure 2:**
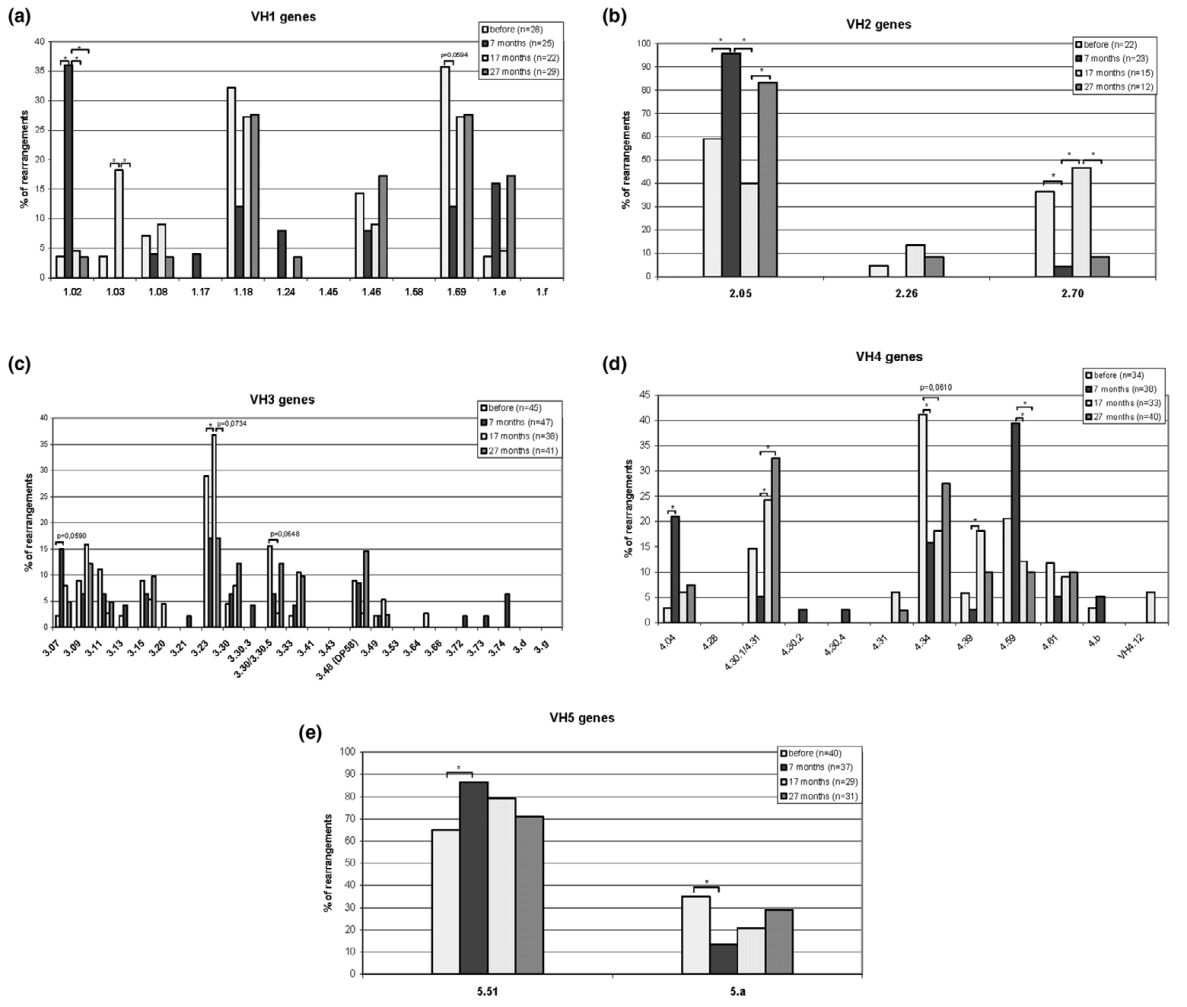
Immunoglobulin heavy-chain gene (Ig-V_H_) distribution in peripheral B cells from patient A at the different time points (0, 7, 17 and 27 months): **(a) **V_H_1 genes, **(b) **V_H_2 genes, **(c) **V_H_3 genes, **(d) **V_H_4 genes and **(e) **V_H_5 genes. Results presented as the percentage of rearrangements expressing one particular gene within one V_H _family. * *P *< 0.05 using Fisher's exact test.

**Table 1:** Rheumatoid factor values at different time points following the first anti-CD20 antibody therapy

	Rheumatoid factor values (U/ml)						
	
	0 months^a^	2 months	5 months	7 months	13 months	17 months	27 months
Patient A	682	295	196	148	186	284	80
Patient B	196		124	130			

^a^Months after first therapy.

**Table 2:** Comparison of the overall mutational frequencies of V_H_ rearrangements obtained from individual peripheral CD19^+ ^(CD27^- ^or CD27^+^) B cells of rheumatoid arthritis patient A before therapy and 7 months after first therapy

	Before therapy				7 months after therapy			
	
	*n*	Mutations (*n*)	Total bp	Mutational frequency (%)	*n*	Mutations (*n*)	Total bp	Mutational frequency (%)
CD19^+^CD27^-^	18	27	4,349	0.62	15	183	3,443	5.31*
CD19^+^CD27^+^	Not determined				22	477	5,745	8.30

* *P *< 0.0001 versus before therapy using chi-square test.
